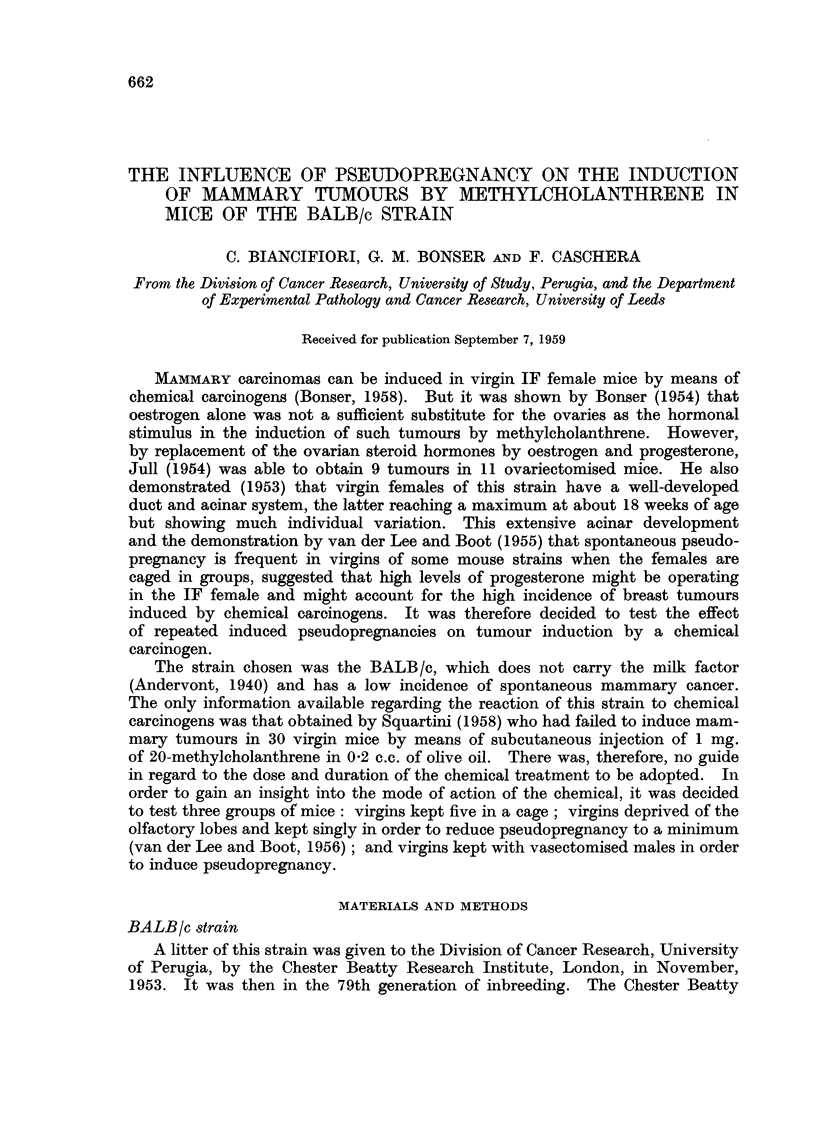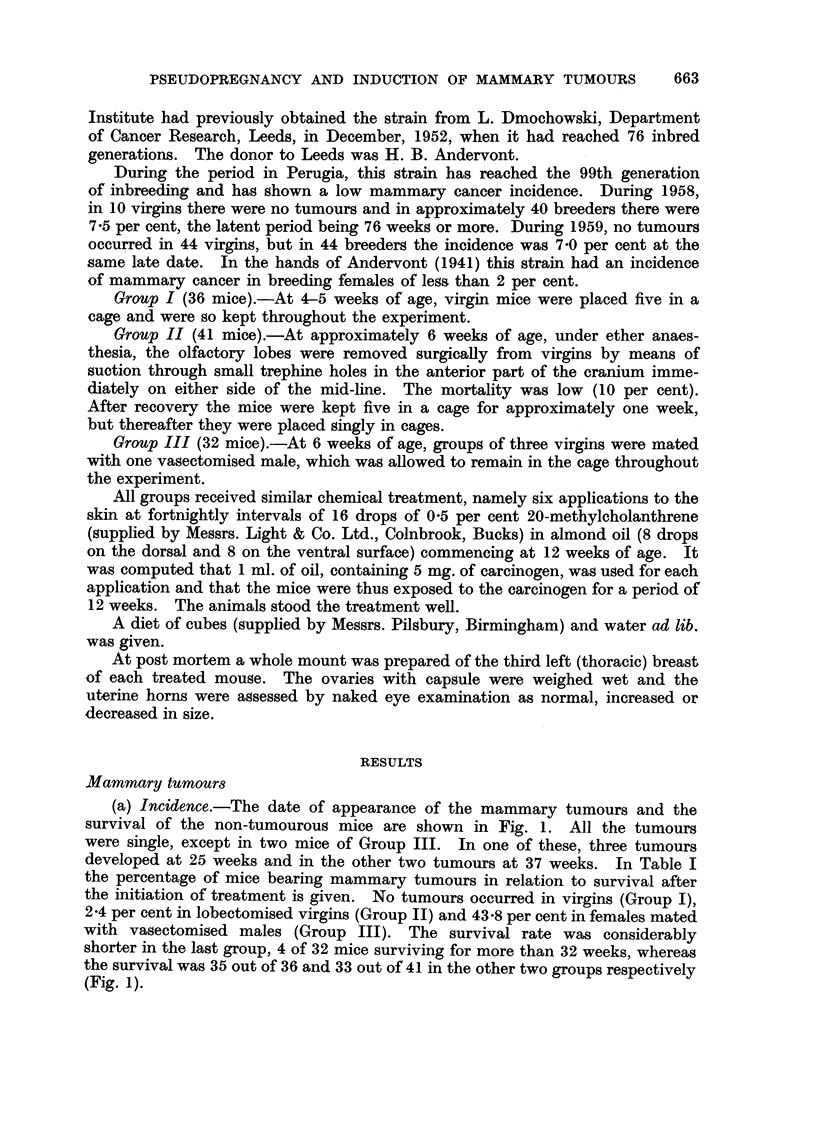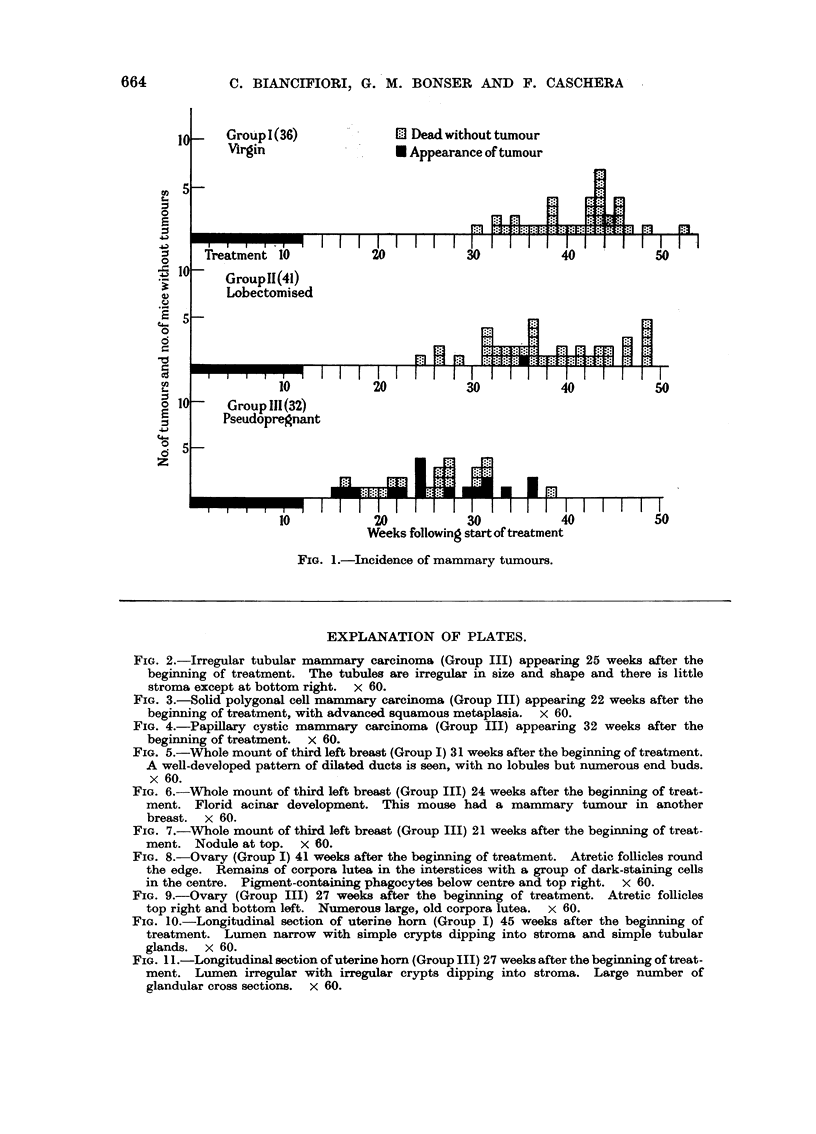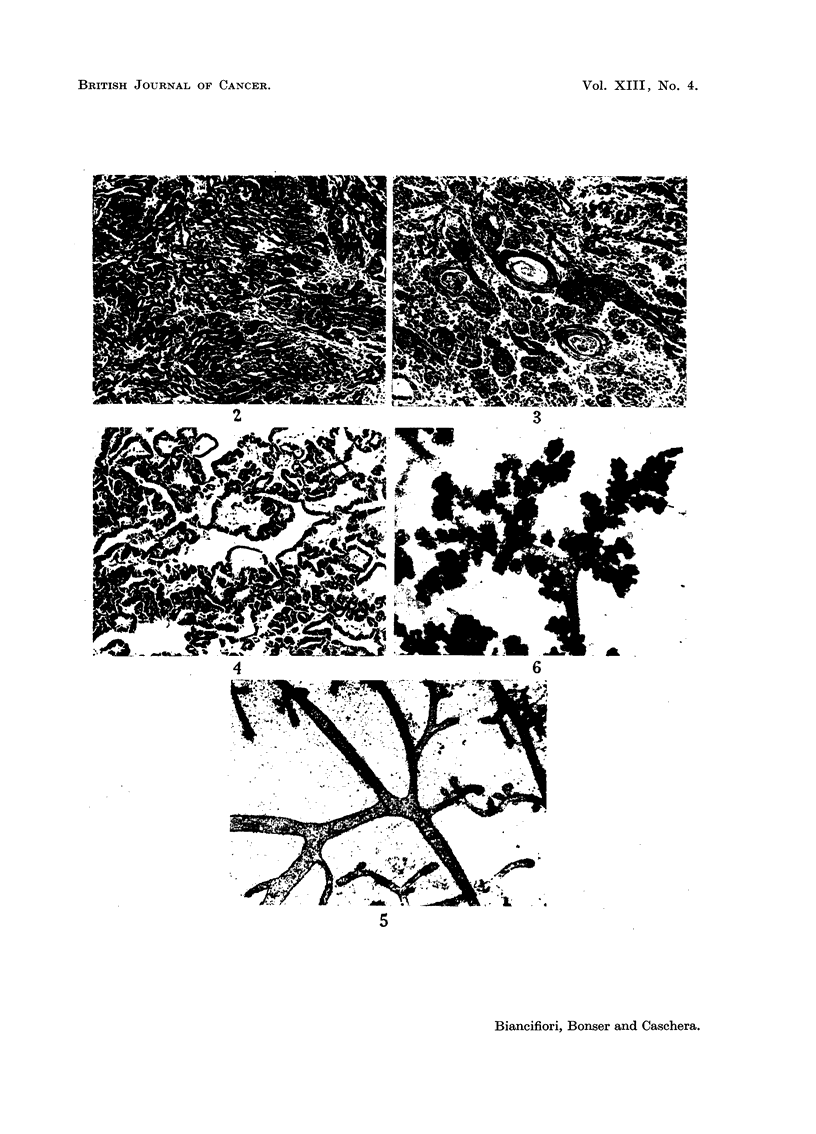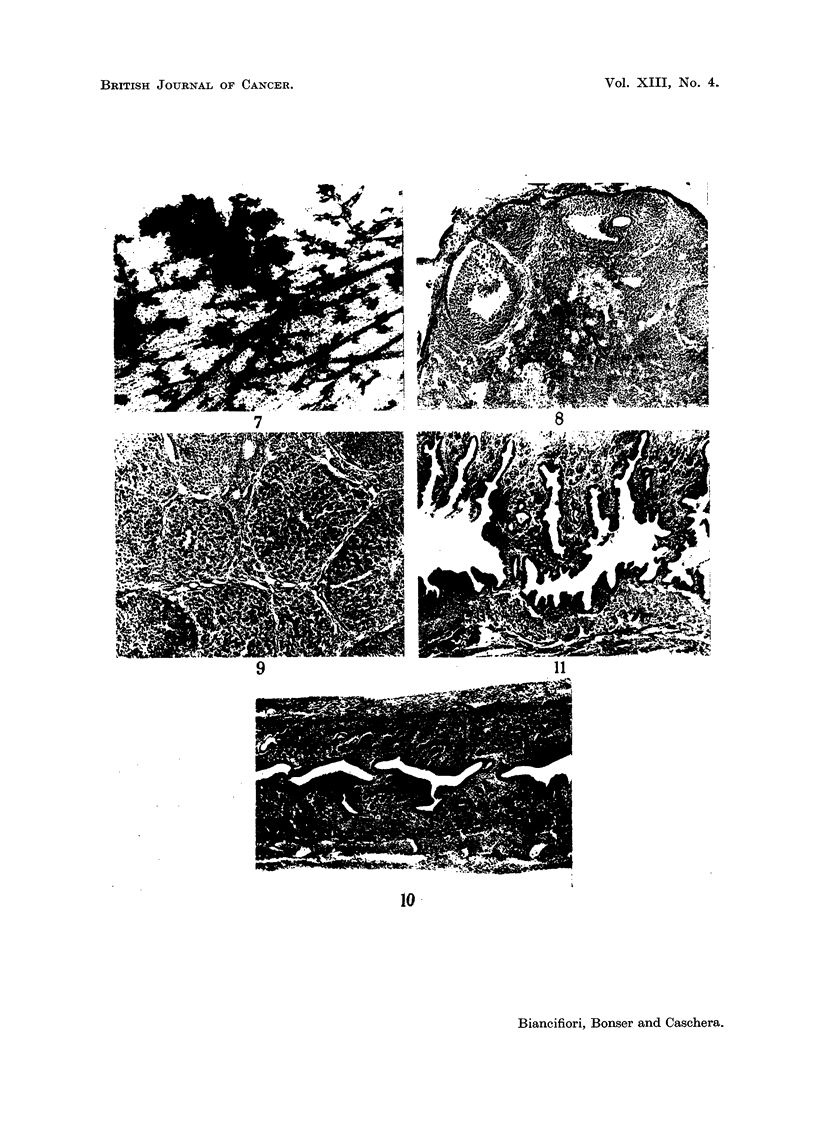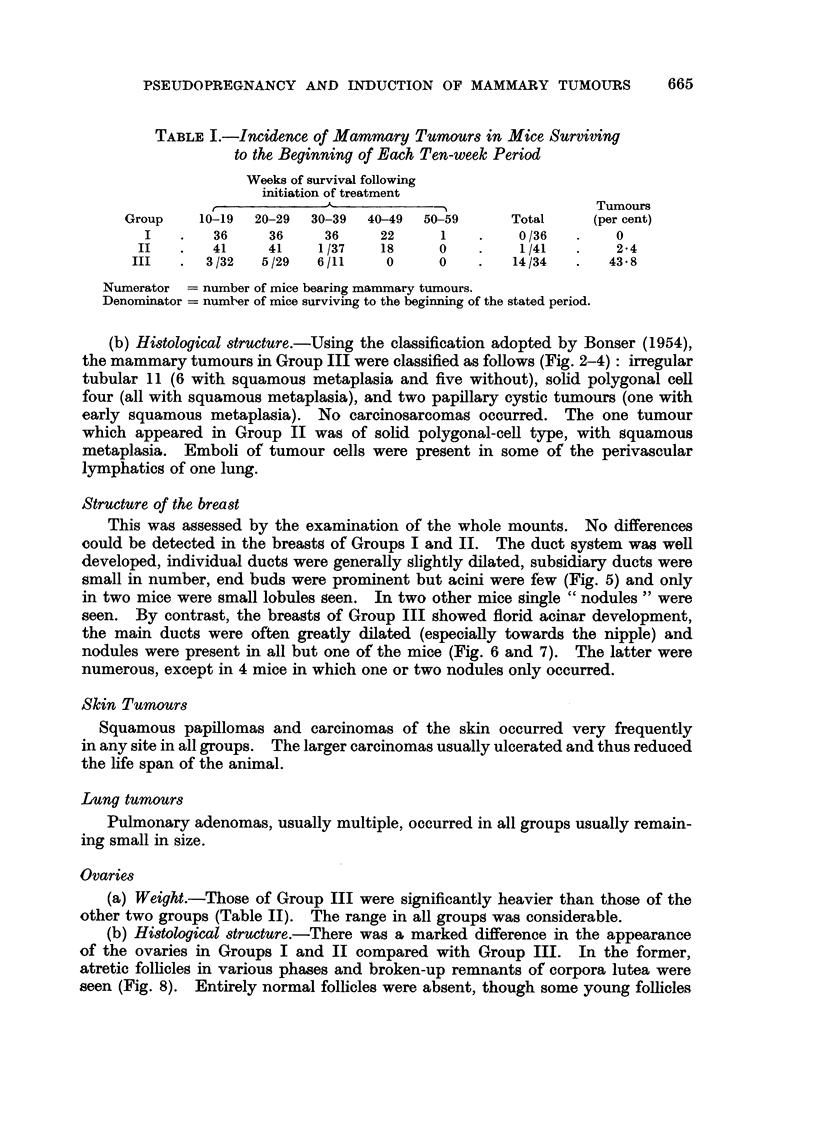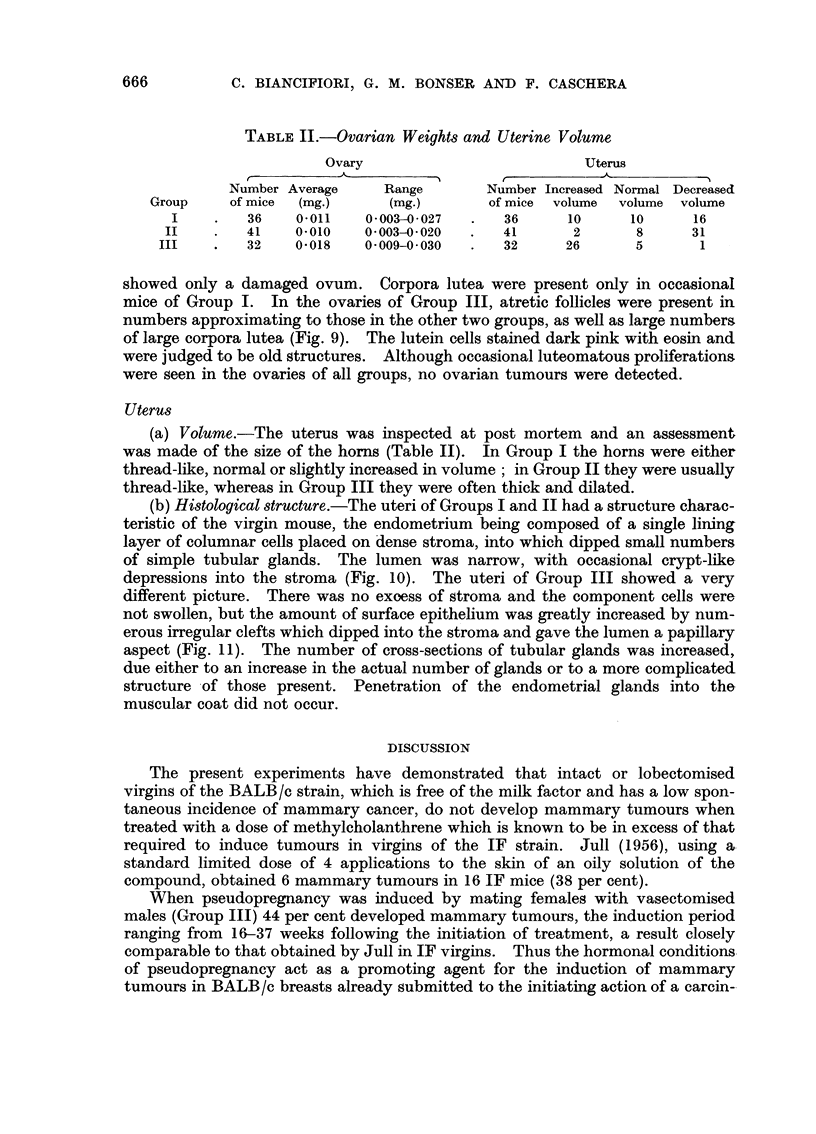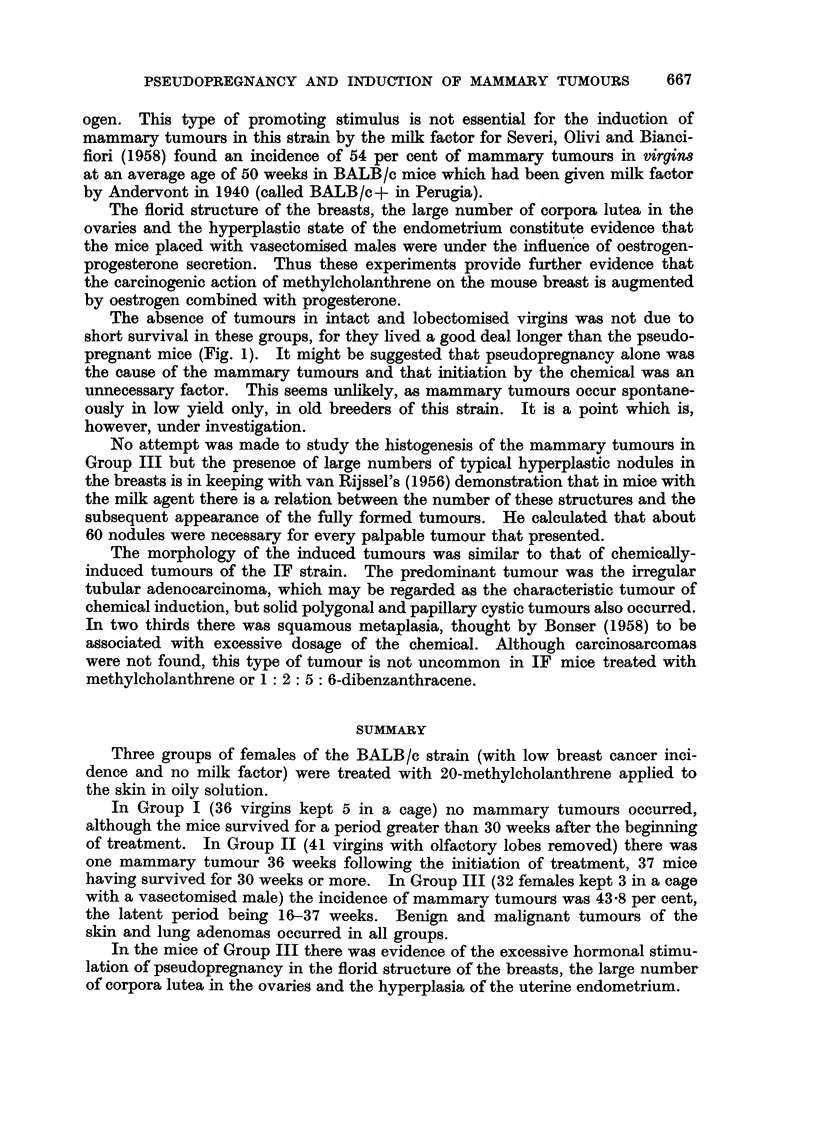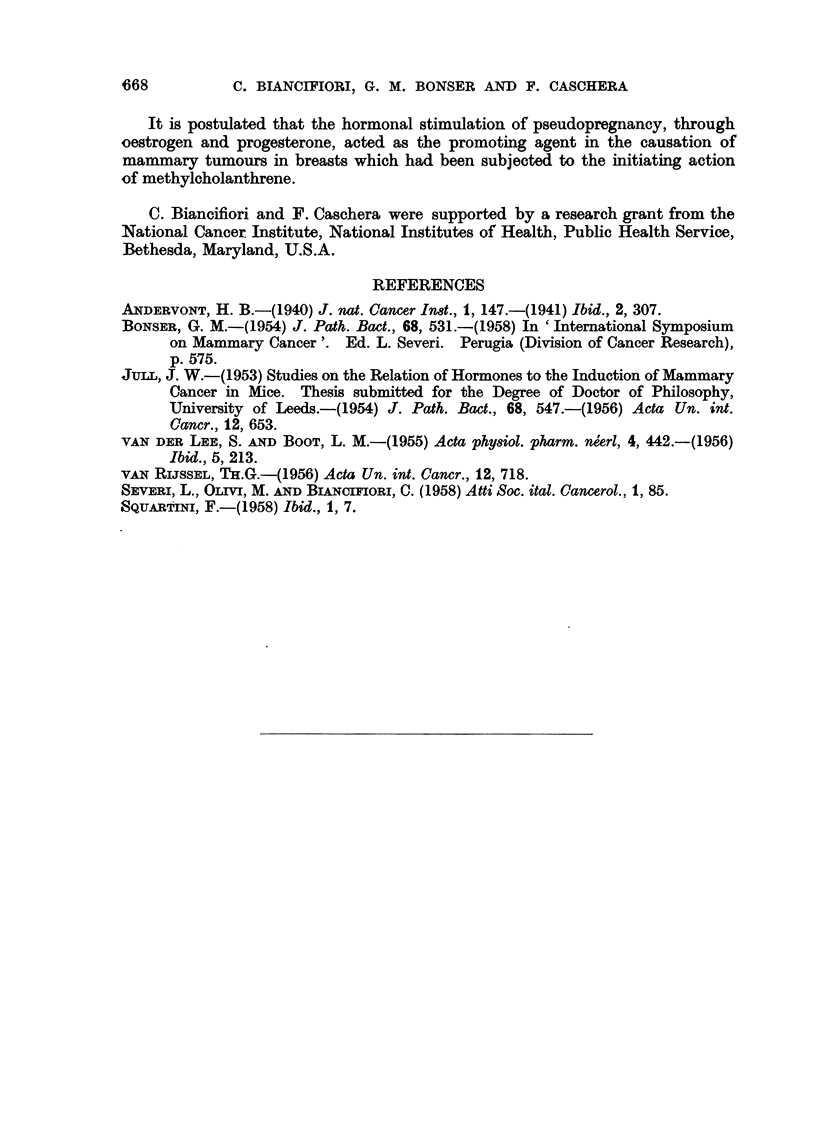# The Influence of Pseudopregnancy on the Induction of Mammary Tumours by Methylcholanthrenein Mice of the Balb/c Strain

**DOI:** 10.1038/bjc.1959.73

**Published:** 1959-12

**Authors:** C. Biancifiori, G. M. Bonser, F. Caschera

## Abstract

**Images:**


### THE INFLUENCE OF PSEUDOPREGNANCY ON THE INDUCTION OF MAMMARY TUMOURS BY METHYLCHOLANTHRENE IN MICE OF THE BALB/c STRAIN

C. BIANCIFIORI, G. M. BONSER AND F. CASCHERA

*From the Division of Cancer Research, University of Study, Perugia, and the Department of Experimental Pathology and Cancer Research, University of Leeds*

Received for publication September 7, 1959

MAMMARY carcinomas can be induced in virgin IF female mice by means of chemical carcinogens (Bonser, 1958). But it was shown by Bonser (1954) that oestrogen alone was not a sufficient substitute for the ovaries as the hormonal stimulus in the induction of such tumours by methylcholanthrene. However, by replacement of the ovarian steroid hormones by oestrogen and progesterone, Jull (1954) was able to obtain 9 tumours in 11 ovariectomised mice. He also demonstrated (1953) that virgin females of this strain have a well-developed duct and acinar system, the latter reaching a maximum at about 18 weeks of age but showing much individual variation. This extensive acinar development and the demonstration by van der Lee and Boot (1955) that spontaneous pseudopregnancy is frequent in virgins of some mouse strains when the females are caged in groups, suggested that high levels of progesterone might be operating in the IF female and might account for the high incidence of breast tumours induced by chemical carcinogens. It was therefore decided to test the effect of repeated induced pseudopregnancies on tumour induction by a chemical carcinogen.

The strain chosen was the BALB/c, which does not carry the milk factor (Andervont, 1940) and has a low incidence of spontaneous mammary cancer. The only information available regarding the reaction of this strain to chemical carcinogens was that obtained by Squartini (1958) who had failed to induce mammary tumours in 30 virgin mice by means of subcutaneous injection of 1 mg. of 20-methylcholanthrene in 0·2 c.c. of olive oil. There was, therefore, no guide in regard to the dose and duration of the chemical treatment to be adopted. In order to gain an insight into the mode of action of the chemical, it was decided to test three groups of mice : virgins kept five in a cage ; virgins deprived of the olfactory lobes and kept singly in order to reduce pseudopregnancy to a minimum (van der Lee and Boot, 1956) ; and virgins kept with vasectomised males in order to induce pseudopregnancy.

### MATERIALS AND METHODS

### *BALB/c strain*

A litter of this strain was given to the Division of Cancer Research, University of Perugia, by the Chester Beatty Research Institute, London, in November, 1953. It was then in the 79th generation of inbreeding. The Chester Beatty

Institute had previously obtained the strain from L. Dmochowski, Department of Cancer Research, Leeds, in December, 1952, when it had reached 76 inbred generations. The donor to Leeds was H. B. Andervont.

During the period in Perugia, this strain has reached the 99th generation of inbreeding and has shown a low mammary cancer incidence. During 1958, in 10 virgins there were no tumours and in approximately 40 breeders there were 7·5 per cent, the latent period being 76 weeks or more. During 1959, no tumours occurred in 44 virgins, but in 44 breeders the incidence was 7·0 per cent at the same late date. In the hands of Andervont (1941) this strain had an incidence of mammary cancer in breeding females of less than 2 per cent.

*Group I* (36 mice).—At 4–5 weeks of age, virgin mice were placed five in a cage and were so kept throughout the experiment.

*Group II* (41 mice).—At approximately 6 weeks of age, under ether anaesthesia, the olfactory lobes were removed surgically from virgins by means of suction through small trephine holes in the anterior part of the cranium immediately on either side of the mid-line. The mortality was low (10 per cent). After recovery the mice were kept five in a cage for approximately one week, but thereafter they were placed singly in cages.

*Group III* (32 mice).—At 6 weeks of age, groups of three virgins were mated with one vasectomised male, which was allowed to remain in the cage throughout the experiment.

All groups received similar chemical treatment, namely six applications to the skin at fortnightly intervals of 16 drops of 0·5 per cent 20-methylcholanthrene (supplied by Messrs. Light & Co. Ltd., Colnbrook, Bucks) in almond oil (8 drops on the dorsal and 8 on the ventral surface) commencing at 12 weeks of age. It was computed that 1 ml. of oil, containing 5 mg. of carcinogen, was used for each application and that the mice were thus exposed to the carcinogen for a period of 12 weeks. The animals stood the treatment well.

A diet of cubes (supplied by Messrs. Pilsbury, Birmingham) and water *ad lib.* was given.

At post mortem a whole mount was prepared of the third left (thoracic) breast of each treated mouse. The ovaries with capsule were weighed wet and the uterine horns were assessed by naked eye examination as normal, increased or decreased in size.

### RESULTS

### *Mammary tumours*

(a) *Incidence.*—The date of appearance of the mammary tumours and the survival of the non-tumourous mice are shown in Fig. 1. All the tumours were single, except in two mice of Group III. In one of these, three tumours developed at 25 weeks and in the other two tumours at 37 weeks. In Table I the percentage of mice bearing mammary tumours in relation to survival after the initiation of treatment is given. No tumours occurred in virgins (Group I), 2·4 per cent in lobectomised virgins (Group II) and 43·8 per cent in females mated with vasectomised males (Group III). The survival rate was considerably shorter in the last group, 4 of 32 mice surviving for more than 32 weeks, whereas the survival was 35 out of 36 and 33 out of 41 in the other two groups respectively (Fig. 1).

FIG. 1.—Incidence of mammary tumours.

---

### EXPLANATION OF PLATES.

FIG. 2.—Irregular tubular mammary carcinoma (Group III) appearing 25 weeks after the beginning of treatment. The tubules are irregular in size and shape and there is little stroma except at bottom right. × 60.

FIG. 3.—Solid polygonal cell mammary carcinoma (Group III) appearing 22 weeks after the beginning of treatment, with advanced squamous metaplasia. × 60.

FIG. 4.—Papillary cystic mammary carcinoma (Group III) appearing 32 weeks after the beginning of treatment. × 60.

FIG. 5.—Whole mount of third left breast (Group I) 31 weeks after the beginning of treatment. A well-developed pattern of dilated ducts is seen, with no lobules but numerous end buds. × 60.

FIG. 6.—Whole mount of third left breast (Group III) 24 weeks after the beginning of treatment. Florid acinar development. This mouse had a mammary tumour in another breast. × 60.

FIG. 7.—Whole mount of third left breast (Group III) 21 weeks after the beginning of treatment. Nodule at top. × 60.

FIG. 8.—Ovary (Group I) 41 weeks after the beginning of treatment. Atretic follicles round the edge. Remains of corpora lutea in the interstices with a group of dark-staining cells in the centre. Pigment-containing phagocytes below centre and top right. × 60.

FIG. 9.—Ovary (Group III) 27 weeks after the beginning of treatment. Atretic follicles top right and bottom left. Numerous large, old corpora lutea. × 60.

FIG. 10.—Longitudinal section of uterine horn (Group I) 45 weeks after the beginning of treatment. Lumen narrow with simple crypts dipping into stroma and simple tubular glands. × 60.

FIG. 11.—Longitudinal section of uterine horn (Group III) 27 weeks after the beginning of treatment. Lumen irregular with irregular crypts dipping into stroma. Large number of glandular cross sections. × 60.

2

3

4

6

5

Biancifiori, Bonser and Caschera.

7

8

9

11

10

TABLE I.—*Incidence of Mammary Tumours in Mice Surviving to the Beginning of Each Ten-week Period*

| Group | Weeks of survival following initiation of treatment: 10–19 | 20–29 | 30–39 | 40–49 | 50–59 | Total | Tumours (per cent) |
|---|---|---|---|---|---|---|---|
| I | 36 | 36 | 36 | 22 | 1 | 0/36 | 0 |
| II | 41 | 41 | 1/37 | 18 | 0 | 1/41 | 2·4 |
| III | 3/32 | 5/29 | 6/11 | 0 | 0 | 14/34 | 43·8 |

Numerator = number of mice bearing mammary tumours.
Denominator = number of mice surviving to the beginning of the stated period.

(b) *Histological structure.*—Using the classification adopted by Bonser (1954), the mammary tumours in Group III were classified as follows (Fig. 2–4) : irregular tubular 11 (6 with squamous metaplasia and five without), solid polygonal cell four (all with squamous metaplasia), and two papillary cystic tumours (one with early squamous metaplasia). No carcinosarcomas occurred. The one tumour which appeared in Group II was of solid polygonal-cell type, with squamous metaplasia. Emboli of tumour cells were present in some of the perivascular lymphatics of one lung.

*Structure of the breast*

This was assessed by the examination of the whole mounts. No differences could be detected in the breasts of Groups I and II. The duct system was well developed, individual ducts were generally slightly dilated, subsidiary ducts were small in number, end buds were prominent but acini were few (Fig. 5) and only in two mice were small lobules seen. In two other mice single " nodules " were seen. By contrast, the breasts of Group III showed florid acinar development, the main ducts were often greatly dilated (especially towards the nipple) and nodules were present in all but one of the mice (Fig. 6 and 7). The latter were numerous, except in 4 mice in which one or two nodules only occurred.

*Skin Tumours*

Squamous papillomas and carcinomas of the skin occurred very frequently in any site in all groups. The larger carcinomas usually ulcerated and thus reduced the life span of the animal.

*Lung tumours*

Pulmonary adenomas, usually multiple, occurred in all groups usually remaining small in size.

*Ovaries*

(a) *Weight.*—Those of Group III were significantly heavier than those of the other two groups (Table II). The range in all groups was considerable.

(b) *Histological structure.*—There was a marked difference in the appearance of the ovaries in Groups I and II compared with Group III. In the former, atretic follicles in various phases and broken-up remnants of corpora lutea were seen (Fig. 8). Entirely normal follicles were absent, though some young follicles

TABLE II.—*Ovarian Weights and Uterine Volume*

| | Ovary | | | Uterus | | | |
|---|---|---|---|---|---|---|---|
| Group | Number of mice | Average (mg.) | Range (mg.) | Number of mice | Increased volume | Normal volume | Decreased volume |
| I | 36 | 0·011 | 0·003–0·027 | 36 | 10 | 10 | 16 |
| II | 41 | 0·010 | 0·003–0·020 | 41 | 2 | 8 | 31 |
| III | 32 | 0·018 | 0·009–0·030 | 32 | 26 | 5 | 1 |

showed only a damaged ovum. Corpora lutea were present only in occasional mice of Group I. In the ovaries of Group III, atretic follicles were present in numbers approximating to those in the other two groups, as well as large numbers of large corpora lutea (Fig. 9). The lutein cells stained dark pink with eosin and were judged to be old structures. Although occasional luteomatous proliferations were seen in the ovaries of all groups, no ovarian tumours were detected.

*Uterus*

(a) *Volume.*—The uterus was inspected at post mortem and an assessment was made of the size of the horns (Table II). In Group I the horns were either thread-like, normal or slightly increased in volume ; in Group II they were usually thread-like, whereas in Group III they were often thick and dilated.

(b) *Histological structure.*—The uteri of Groups I and II had a structure characteristic of the virgin mouse, the endometrium being composed of a single lining layer of columnar cells placed on dense stroma, into which dipped small numbers of simple tubular glands. The lumen was narrow, with occasional crypt-like depressions into the stroma (Fig. 10). The uteri of Group III showed a very different picture. There was no excess of stroma and the component cells were not swollen, but the amount of surface epithelium was greatly increased by numerous irregular clefts which dipped into the stroma and gave the lumen a papillary aspect (Fig. 11). The number of cross-sections of tubular glands was increased, due either to an increase in the actual number of glands or to a more complicated structure of those present. Penetration of the endometrial glands into the muscular coat did not occur.

### DISCUSSION

The present experiments have demonstrated that intact or lobectomised virgins of the BALB/c strain, which is free of the milk factor and has a low spontaneous incidence of mammary cancer, do not develop mammary tumours when treated with a dose of methylcholanthrene which is known to be in excess of that required to induce tumours in virgins of the IF strain. Jull (1956), using a standard limited dose of 4 applications to the skin of an oily solution of the compound, obtained 6 mammary tumours in 16 IF mice (38 per cent).

When pseudopregnancy was induced by mating females with vasectomised males (Group III) 44 per cent developed mammary tumours, the induction period ranging from 16–37 weeks following the initiation of treatment, a result closely comparable to that obtained by Jull in IF virgins. Thus the hormonal conditions of pseudopregnancy act as a promoting agent for the induction of mammary tumours in BALB/c breasts already submitted to the initiating action of a carcinogen. This type of promoting stimulus is not essential for the induction of mammary tumours in this strain by the milk factor for Severi, Olivi and Biancifiori (1958) found an incidence of 54 per cent of mammary tumours in *virgins* at an average age of 50 weeks in BALB/c mice which had been given milk factor by Andervont in 1940 (called BALB/c+ in Perugia).

The florid structure of the breasts, the large number of corpora lutea in the ovaries and the hyperplastic state of the endometrium constitute evidence that the mice placed with vasectomised males were under the influence of oestrogen-progesterone secretion. Thus these experiments provide further evidence that the carcinogenic action of methylcholanthrene on the mouse breast is augmented by oestrogen combined with progesterone.

The absence of tumours in intact and lobectomised virgins was not due to short survival in these groups, for they lived a good deal longer than the pseudopregnant mice (Fig. 1). It might be suggested that pseudopregnancy alone was the cause of the mammary tumours and that initiation by the chemical was an unnecessary factor. This seems unlikely, as mammary tumours occur spontaneously in low yield only, in old breeders of this strain. It is a point which is, however, under investigation.

No attempt was made to study the histogenesis of the mammary tumours in Group III but the presence of large numbers of typical hyperplastic nodules in the breasts is in keeping with van Rijssel's (1956) demonstration that in mice with the milk agent there is a relation between the number of these structures and the subsequent appearance of the fully formed tumours. He calculated that about 60 nodules were necessary for every palpable tumour that presented.

The morphology of the induced tumours was similar to that of chemically-induced tumours of the IF strain. The predominant tumour was the irregular tubular adenocarcinoma, which may be regarded as the characteristic tumour of chemical induction, but solid polygonal and papillary cystic tumours also occurred. In two thirds there was squamous metaplasia, thought by Bonser (1958) to be associated with excessive dosage of the chemical. Although carcinosarcomas were not found, this type of tumour is not uncommon in IF mice treated with methylcholanthrene or 1 : 2 : 5 : 6-dibenzanthracene.

### SUMMARY

Three groups of females of the BALB/c strain (with low breast cancer incidence and no milk factor) were treated with 20-methylcholanthrene applied to the skin in oily solution.

In Group I (36 virgins kept 5 in a cage) no mammary tumours occurred, although the mice survived for a period greater than 30 weeks after the beginning of treatment. In Group II (41 virgins with olfactory lobes removed) there was one mammary tumour 36 weeks following the initiation of treatment, 37 mice having survived for 30 weeks or more. In Group III (32 females kept 3 in a cage with a vasectomised male) the incidence of mammary tumours was 43·8 per cent, the latent period being 16–37 weeks. Benign and malignant tumours of the skin and lung adenomas occurred in all groups.

In the mice of Group III there was evidence of the excessive hormonal stimulation of pseudopregnancy in the florid structure of the breasts, the large number of corpora lutea in the ovaries and the hyperplasia of the uterine endometrium.

It is postulated that the hormonal stimulation of pseudopregnancy, through oestrogen and progesterone, acted as the promoting agent in the causation of mammary tumours in breasts which had been subjected to the initiating action of methylcholanthrene.

C. Biancifiori and F. Caschera were supported by a research grant from the National Cancer Institute, National Institutes of Health, Public Health Service, Bethesda, Maryland, U.S.A.

### REFERENCES

ANDERVONT, H. B.—(1940) *J. nat. Cancer Inst.*, **1**, 147.—(1941) *Ibid.*, **2**, 307.

BONSER, G. M.—(1954) *J. Path. Bact.*, **68**, 531.—(1958) In 'International Symposium on Mammary Cancer'. Ed. L. Severi. Perugia (Division of Cancer Research), p. 575.

JULL, J. W.—(1953) Studies on the Relation of Hormones to the Induction of Mammary Cancer in Mice. Thesis submitted for the Degree of Doctor of Philosophy, University of Leeds.—(1954) *J. Path. Bact.*, **68**, 547.—(1956) *Acta Un. int. Cancr.*, **12**, 653.

VAN DER LEE, S. AND BOOT, L. M.—(1955) *Acta physiol. pharm. néerl*, **4**, 442.—(1956) *Ibid.*, **5**, 213.

VAN RIJSSEL, TH.G.—(1956) *Acta Un. int. Cancr.*, **12**, 718.

SEVERI, L., OLIVI, M. AND BIANCIFIORI, C. (1958) *Atti Soc. ital. Cancerol.*, **1**, 85.

SQUARTINI, F.—(1958) *Ibid.*, **1**, 7.